# Prostate cancer cell proliferation is influenced by LDL-cholesterol availability and cholesteryl ester turnover

**DOI:** 10.1186/s40170-021-00278-1

**Published:** 2022-01-15

**Authors:** Nikki L. Raftopulos, Tinashe C. Washaya, Andreas Niederprüm, Antonia Egert, Mariam F. Hakeem-Sanni, Bianca Varney, Atqiya Aishah, Mariya L. Georgieva, Ellinor Olsson, Diandra Z. dos Santos, Zeyad D. Nassar, Blake J. Cochran, Shilpa R. Nagarajan, Meghna S. Kakani, Jordan F. Hastings, David R. Croucher, Kerry-Anne Rye, Lisa M. Butler, Thomas Grewal, Andrew J. Hoy

**Affiliations:** 1 School of Medical Sciences, Charles Perkins Centre, Faculty of Medicine and Health, The University of Sydney, Sydney, New South Wales Australia; 2 School of Pharmacy, Faculty of Medicine and Health, The University of Sydney, Sydney, New South Wales Australia; 3 Faculty of Medicine, Ruprecht Karl University of Heidelberg, Baden-Wuerttemberg, Heidelberg, Germany; 4 Biotechnology Program/RENORBIO, Health Sciences Center, Federal University of Espirito Santo, Vitoria, ES Brazil; 5 Adelaide Medical School and Freemasons Centre for Male Health and Wellbeing, University of Adelaide, Adelaide, South Australia Australia; 6 South Australian Health and Medical Research Institute, Adelaide, South Australia Australia; 7 School of Medical Sciences, Faculty of Medicine, University of New South Wales, Sydney, New South Wales Australia; 8 The Kinghorn Cancer Centre, Garvan Institute of Medical Research, Sydney, New South Wales Australia; 9 St Vincent’s Hospital Clinical School, Faculty of Medicine, University of New South Wales, Sydney, New South Wales Australia

**Keywords:** Prostate cancer, LDL, LDL-cholesterol, Cell proliferation, Cholesteryl ester, ACAT1, nCEH1, HSL

## Abstract

**Background:**

Prostate cancer growth is driven by androgen receptor signaling, and advanced disease is initially treatable by depleting circulating androgens. However, prostate cancer cells inevitably adapt, resulting in disease relapse with incurable castrate-resistant prostate cancer. Androgen deprivation therapy has many side effects, including hypercholesterolemia, and more aggressive and castrate-resistant prostate cancers typically feature cellular accumulation of cholesterol stored in the form of cholesteryl esters. As cholesterol is a key substrate for de novo steroidogenesis in prostate cells, this study hypothesized that castrate-resistant/advanced prostate cancer cell growth is influenced by the availability of extracellular, low-density lipoprotein (LDL)-derived, cholesterol, which is coupled to intracellular cholesteryl ester homeostasis.

**Methods:**

C4-2B and PC3 prostate cancer cells were cultured in media supplemented with fetal calf serum (FCS), charcoal-stripped FCS (CS-FCS), lipoprotein-deficient FCS (LPDS), or charcoal-stripped LPDS (CS-LPDS) and analyzed by a variety of biochemical techniques. Cell viability and proliferation were measured by MTT assay and Incucyte, respectively.

**Results:**

Reducing lipoprotein availability led to a reduction in cholesteryl ester levels and cell growth in C4-2B and PC3 cells, with concomitant reductions in PI3K/mTOR and p38MAPK signaling. This reduced growth in LPDS-containing media was fully recovered by supplementation of exogenous low-density lipoprotein (LDL), but LDL only partially rescued growth of cells cultured with CS-LPDS. This growth pattern was not associated with changes in androgen receptor signaling but rather increased p38MAPK and MEK1/ERK/MSK1 activation. The ability of LDL supplementation to rescue cell growth required cholesterol esterification as well as cholesteryl ester hydrolysis activity. Further, growth of cells cultured in low androgen levels (CS-FCS) was suppressed when cholesteryl ester hydrolysis was inhibited.

**Conclusions:**

Overall, these studies demonstrate that androgen-independent prostate cancer cell growth can be influenced by extracellular lipid levels and LDL-cholesterol availability and that uptake of extracellular cholesterol, through endocytosis of LDL-derived cholesterol and subsequent delivery and storage in the lipid droplet as cholesteryl esters, is required to support prostate cancer cell growth. This provides new insights into the relationship between extracellular cholesterol, intracellular cholesterol metabolism, and prostate cancer cell growth and the potential mechanisms linking hypercholesterolemia and more aggressive prostate cancer.

## Background

The progression of prostate cancer, and other solid tumors, is supported by changes in cancer cell metabolism that are geared towards increasing biomass synthesis. One critical component is covering the increased demand for lipids in cellular membranes during proliferation [1], in particular cholesterol as it is an essential constituent of cellular membranes, comprising up to 30% of lipid content. Cholesterol metabolism in prostate cancer has received significant attention in recent years (see reviews [2, 3]). Beyond the role of cholesterol metabolism in oncogenesis and the differences in cholesterol biology observed between normal tissue and tumor, cholesterol metabolism has been suggested to play key roles in other aspects of prostate cancer pathophysiology including treatment resistance [2, 4].

Androgen deprivation therapy has remained the frontline strategy for clinical management of locally-recurrent and/or metastatic disease due to the dependence of prostate cancer cells on androgens for growth and survival. Although androgen deprivation therapy is initially successful in slowing prostate cancer progression, patients inevitably develop lethal castrate-resistant disease (CRPC), due to the emergence of adaptive survival pathways that reprogram androgen signaling and/or activate alternative tumor survival pathways [5]. Androgen deprivation therapy, by creating a low androgen environment, induces pronounced systemic metabolic changes including hypercholesterolemia [6], which may result in a plentiful supply of cholesterol for de novo steroidogenesis as an adaptive mechanism to promote the development of CRPC [7]. In fact, hypercholesterolemia is associated with a shorter time to the development of CRPC in patients who have undergone androgen deprivation therapy [8]. Several studies have also shown a relationship between elevated circulating cholesterol levels and a higher risk of prostate cancer development and progression [9–12]. Conversely, patients who use cholesterol-lowering agents such as statins have a lower risk of advanced prostate cancer and reduced prostate cancer-specific mortality (see reviews [13, 14]); however, these associations are somewhat controversial [15].

The cellular levels of cholesterol are normally tightly regulated through the balance of uptake, de novo synthesis and storage in lipid droplets (LDs) as cholesteryl esters, notably via the feedback control mechanism between de novo synthesis and uptake whereby high intracellular levels of cholesterol lead to decreased expression of low-density lipoprotein receptor (LDLR) to reduce uptake of extracellular LDL-derived cholesterol. In prostate, and other cancers, this feedback loop is disrupted, and despite ongoing de novo cholesterol synthesis, increased levels of LDLR are observed, thereby elevating uptake of cholesterol and essential fatty acids to support cell growth and survival [16]. Consistent with this concept, pharmacologically targeting cholesteryl ester synthesis by blocking acyl-Coenzyme A: cholesterol acyltransferase 1 (ACAT1) activity impaired androgen-independent, androgen receptor (AR)-negative PC3 prostate cancer cell growth [17]. Specifically, reduced ACAT1-catalyzed synthesis of cholesteryl esters led to free cholesterol accumulation, lowered LDLR levels, and reduced essential fatty acid uptake to impair cell proliferation and in vivo tumor growth [17]. As such, this study implicated storage of cholesteryl esters in LDs as an important process that supports AR-negative PC3 prostate cancer cell viability. LDs serve as temporary storage sites for cholesterol and so may influence AR-positive prostate cancer cell biology via other mechanisms. For example, steroidogenic tissues, such as the adrenals and gonads, accumulate cholesteryl ester-rich LDs and this intracellular source of cholesterol serves as a primary substrate for steroidogenesis in these tissues [18]. Importantly, prostate cancer tissues accumulate LDs that are cholesteryl ester-rich, whereas normal prostate tissue and benign prostate hyperplasia have virtually no visible LDs [17]. Moreover, high-grade localized prostate cancer and metastatic cancer are characterized by an increased LD content compared to low-grade localized cancer [17]. As such, this suggests that the increase in cholesteryl esters may serve as an important source of cholesterol, via the actions of cholesteryl ester hydrolases, for de novo steroidogenesis and thereby influence AR-positive, androgen-independent (i.e., CRPC) prostate cancer cell growth. Based on these findings, we hypothesized that cholesteryl ester hydrolase activity promotes the emergence and growth of CRPC and other treatment-resistant forms of prostate cancer, a concept that has not been explored previously.

## Methods

### Cell culture

The human prostate carcinoma cell lines C4-2B (AR-positive, androgen-independent) and PC3 (AR-negative, androgen-independent) were obtained from the American Type Culture Collection (ATCC). Cell lines were validated periodically by Garvan Molecular Genetics using a test based on the Powerplex 18D kit (DC1808, Promega) and tested for mycoplasma every 3 months (MycoAlert™ mycoplasma detection kit, Lonza). All cell lines were cultured in Roswell Park Memorial Institute (RPMI) 1640 medium (Life Technologies Australia Pty Ltd., Scoresby VIC, Australia) supplemented with 10% fetal calf serum (FCS; HyClone, GE Healthcare Life Sciences, USA) and 100 IU/ml penicillin and 100 IU/ml streptomycin (Life Technologies Australia Pty Ltd., Scoresby VIC, Australia).

LDL was isolated from donated, pooled blood samples from normal healthy donors (obtained from Red Cross, Sydney, Australia; by ultracentrifugation in the 1.019–1.055 g/ml density range) [19]. Lipoprotein-deficient fetal calf serum (LPDS) was prepared by preparative ultracentrifugation [20]. Before experiments, LDL and LPDS were dialyzed extensively against PBS and stored at 4 °C until use. LDL protein concentration was determined by the bicinchoninic acid method (Bio-Rad) [20].

For the charcoal stripping of FCS and LPDS, dextran-coated charcoal (0.5 % activated charcoal, 55 mM dextran, 20 % glycerol) was dissolved in Tris/EDTA buffer (10 mM Tris, 1.5 mM EDTA, 10 mM sodium molybdate, 10% glycerol, pH 7.4) overnight, then centrifuged at 4000 rpm for 30 min using an Allegra X12R centrifuge (Beckman Coulter). The supernatant was removed, and FCS or LPDS added to the charcoal pellet, mixed for 2 h, and then centrifuged at 4000 rpm for 30 min. The serum supernatant was mixed with a charcoal pellet for 2 h, centrifuged, filtered using filter paper (Whatman #41 round filter papers), and then filtration sterilized. Sterilized charcoal-stripped FCS (CS-FCS) and charcoal-stripped LPDS (CS-LPDS) were stored in 4°C until use.

To inhibit ACAT1, cells were treated with Avasimibe (#18129, Cayman Chemical) in RPMI-media, 10% LPDS, 1% Pen/Strep, and LDL (50 μg/ml). To inhibit neutral cholesterol ester hydrolase 1 (nCEH1) activity in C4-2B cells, cells were treated with 1 μM JW480 (#10879, Cayman Chemical) in RPMI, 10% LPDS, LDL (50 μg/ml), and 1% Pen/Strep. To inhibit nCEH1 and hormone-sensitive lipase (HSL) activity in PC3 cells, cells were treated with 1 μM JW480 (#10879, Cayman Chemical) and 1 μM 76-0079 (generous gift from Novo Nordisk) in RPMI, 10% LPDS, LDL (50 μg/ml), and 1% Pen/Strep. In some experiments, cells were cultured with CS-FCS-containing medium supplemented with 1 nM dihydrotestosterone (DHT), which was replenished every 24 h during proliferation assays. In other experiments, cells were treated with 100 nM insulin (Sigma) and 1 μM isoprenaline (Sigma) for 30 min and 60 min in RPMI containing 0.3% BSA.

### Cell proliferation

MTT assays were performed as described previously [21]. Alternatively, the percent cell confluence was continuously measured using IncuCyte-ZOOM according to the manufacturer’s instructions (Essen Bioscience).

### Biochemical procedures

Cellular lipids were extracted using the method of Folch et al. [22] and cholesteryl esters were quantified using an enzymatic Amplex Red® Cholesterol kit (Thermo Fisher Scientific) [23]. Cell protein content was determined using Pierce Micro BCA protein assay (Life Technologies Australia Pty Ltd., Scoresby VIC, Australia). Media cholesterol was measured using the Amplex Red® Cholesterol kit according to manufacturer’s instructions (Thermo Fisher Scientific) [23]. Media testosterone was determined by the Department of Chemical Pathology, Royal Prince Alfred Hospital (Sydney, NSW, Australia). Neutral cholesterol ester hydrolase activity was determined as previously described [24].

### Protein analysis

Protein extraction from cultured cells was performed as described previously [25]. Cell lysates were subjected to SDS-PAGE, transferred to PVDF membranes (Merck Millipore), and then immunoblotted with antibodies against AR (rabbit monoclonal; Cell Signaling #5153S), prostate-specific antigen (PSA; Protein Tech #10679-AP), nCEH1 (rabbit polyclonal; Sigma-Aldrich #SAB4301148), HSL (rabbit monoclonal; Cell Signaling #4107S), glyceraldehyde 3-phosphate dehydrogenase (GAPDH; rabbit monoclonal; Cell Signaling #2118S), and alpha-tubulin (mouse monoclonal; Abcam #ab7291). Chemiluminescence was performed using Luminata Crescendo Western HRP Substrate (Merck Millipore) and imaged using the Bio-Rad ChemiDoc MP Imaging System (Bio-Rad laboratories, Hercules, CA, USA) and Image Lab software 4.1 (Bio-Rad laboratories, Hercules, CA, USA).

Cell lysates were also used within bead-based, multiplex phosphoprotein assays on the Bio-Plex MAGPIX system (BioRad #171015044), as previously described [26]. Both the MILLIPLEX MAPK/SAPK Signaling 10-Plex Kit (Millipore 48-660MAG) and MILLIPLEX Akt/mTOR Phosphoprotein Magnetic Bead 11-Plex Kit (Millipore 8-611MAG) were used, according to the manufacturer’s instructions.

### Dataset analysis

Expression of *ACAT1*, *NCEH1*, and *LIPE* and the corresponding progression-free and overall survival data for prostate cancer were retrieved from The Cancer Genome Atlas (TCGA) data portal, cBioPortal [27, 28], and GEO under accession number GSE35988 and GSE16560. Proteomics data were downloaded from Iglesias-Gato et al. [29].

### Statistical analysis

Statistical analyses were performed with Graphpad Prism 9.2.0 (Graphpad Software, San Diego, CA). Differences among groups were assessed with appropriate statistical tests noted in figure legends. *P* ≤ 0.05 was considered significant. Data are reported as mean ± SEM of at least 3 independent determinations.

## Results

### Reduced availability of androgens does not alter C4-2B and PC3 prostate cancer cell proliferation nor cellular cholesteryl ester levels

Firstly, we assessed the influence of extracellular androgen levels on proliferation of human prostate cancer cell lines. For this, we charcoal-stripped FCS (CS-FCS), which lowered testosterone by 75%, but not cholesterol, compared to FCS (Figure S1A–B), consistent with a previous report [30]. As expected, when cultured in CS-FCS, proliferation of androgen-independent C4-2B (AR-positive) and PC3 (AR-negative) cells was unaffected and comparable to cells grown in FCS-containing media (Figure S1C–D). The reduced availability of serum testosterone to cells cultured in CS-FCS did not alter cellular cholesteryl ester levels in these cells compared to the controls (Figure S1E–F).

### Lipoprotein-deficient serum impairs C4-2B and PC3 cell proliferation and reduces cellular cholesteryl ester levels

**Fig. 1:**
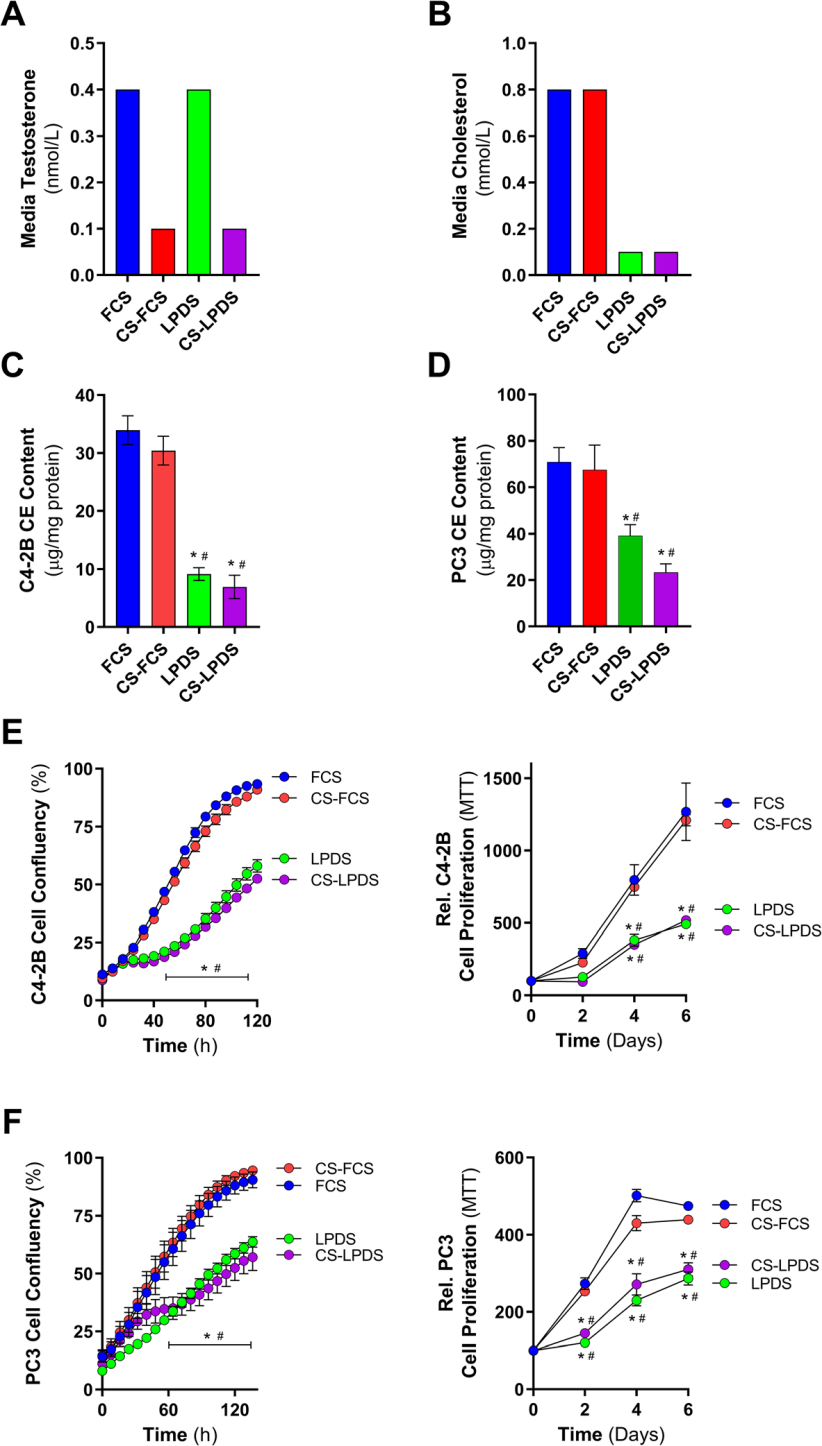
C4-2B and PC3 cell cholesteryl ester and growth are influenced by extracellular cholesterol levels but not androgen levels. **a** Testosterone and **b** cholesterol levels of fetal calf serum (FCS), charcoal-stripped FCS (CS-FCS), lipoprotein-deficient fetal calf serum (LPDS), and charcoal-stripped LPDS (CS-LPDS). **c** C4-2B and **d** PC3 cholesteryl ester levels following 24 h culturing in media supplemented with 10% FCS, 10% CS-FCS, 10% LPDS, or 10% CS-LPDS. **e** C4-2B cell proliferation in media supplemented with 10% FCS, 10% CS-FCS, 10% LPDS, or 10% CS-LPDS determined by IncuCyte and MTT. **f** PC3 cell proliferation in media supplemented with 10% FCS, 10% CS-FCS, 10% LPDS, or 10% CS-LPDS determined by IncuCyte and MTT. Data are presented as mean ± SEM of at least three independent experiments performed in triplicate. * *P* ≤ 0.05 vs. FCS; # *P* ≤ 0.05 vs. CS-FCS by one-way ANOVA (**c** and **d**) or two-way ANOVA (**e** and **f**) followed by Tukey’s multiple comparisons test

Since testosterone levels in the media did not alter cell proliferation, we next assessed the influence of extracellular lipids, in standard or low androgen growth conditions, on C4-2B and PC3 cell proliferation. To achieve this, we prepared lipoprotein-deficient FCS (LPDS), which contained the same amount of testosterone (Fig. 1a), but lower cholesterol levels (~90%), as compared to FCS (Fig. 1b). Further, we prepared lipoprotein-deficient CS-FCS (CS-LPDS) with low levels of both testosterone and cholesterol (Fig. 1a and b). In line with previous findings [17], C4-2B (Fig. 1c) and PC3 (Fig. 1d) cells cultured in LPDS and CS-LPDS contained reduced amounts of cholesteryl ester. Moreover, both cell lines grew slower compared to cells cultured in FCS and CS-FCS, respectively (Fig. 1e and f). Importantly, DHT stimulation, which activated AR signaling in C4-2B cells (Figure S2A), did not restore C4-2B cell growth in CS-LPDS-containing media (Figure S2B). Collectively, these data demonstrate that both AR-positive C4-2B and AR-negative PC3 cell growths are compromised in a lipoprotein- and cholesterol-deficient environment.

### LDL supplementation of LPDS increases cellular cholesteryl ester levels and restores cell proliferation to similar levels as cells cultured in FCS

**Fig. 2:**
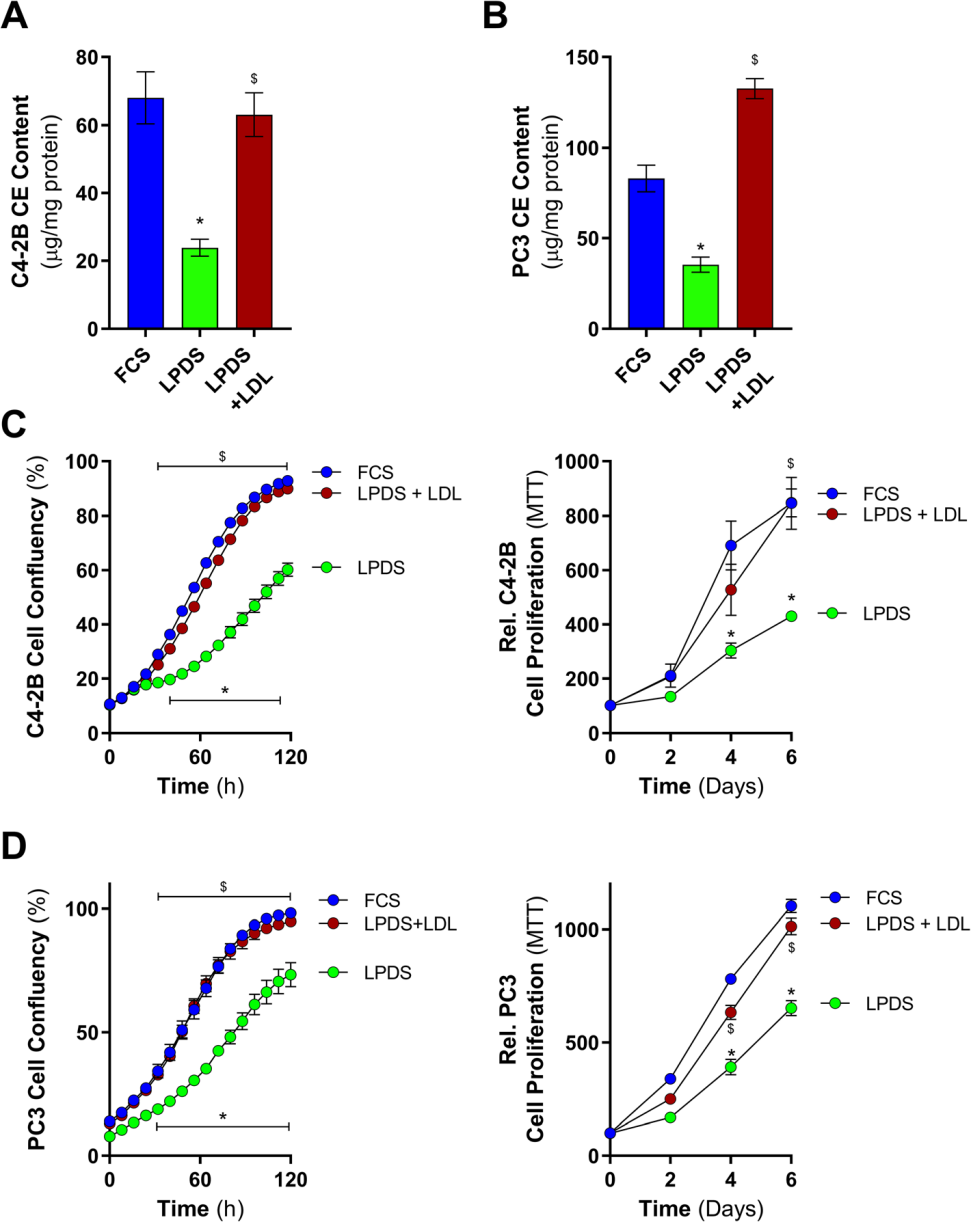
LDL supplementation rescues C4-2B and PC3 cell growth in LPDS-containing media. **a** C4-2B and **b** PC3 cholesteryl ester levels following 24 h culturing in media supplemented with 10% FCS, 10% LPDS, or 10% LPDS plus 50 μg/ml of human LDL. **c** C4-2B cell proliferation cultured in media containing 10% FCS, 10% LPDS, or 10% LPDS plus 50 μg/ml of human LDL by IncuCyte and MTT. **d** PC3 cell proliferation cultured in media containing 10% FCS, 10% LPDS, or 10% LPDS plus 50 μg/ml of human LDL by IncuCyte and MTT. Data are presented as mean ± SEM of at least three independent experiments performed in triplicate. * *P* ≤ 0.05 vs. FCS; $ *P* ≤ 0.05 vs. LPDS by one-way ANOVA (**a** and **b**) or two-way ANOVA (**c** and **d**) followed by Tukey’s multiple comparisons test

The reduced growth of prostate cancer cells cultured in lipoprotein-deficient media, irrespective of androgen levels, and its correlation with reduced cellular cholesteryl ester levels, suggested that cellular uptake of extracellular cholesterol was essential for proliferation. Therefore, to provide an extracellular source of cholesterol, C4-2B and PC3 were cultured in LPDS-containing media alone or supplemented with LDL (50 μg/ml) and, after 24h, cholesteryl ester levels were determined. As expected, cells cultured in LPDS contained lower levels of cholesteryl esters compared to FCS-incubated cells (Fig. 2a and b). LDL supplementation of LPDS-containing media restored cellular cholesteryl ester content to levels comparable to FCS-cultured C4-2B and PC3 cells (Fig. 2a and b). Importantly, LDL-induced restoration of cellular cholesteryl ester levels also restored proliferation of both cell lines to similar levels as when cultured in FCS (Fig. 2a and d). Similar trends were observed when comparing cholesteryl ester levels and growth in cells grown in CS-LPDS with or without LDL, with LDL supplementation restoring cholesteryl ester levels and partially rescuing cell growth (Figure S3). Combined, these data demonstrate that C4-2B and PC3 cell growth can be influenced by extracellular lipids, such as LDL-cholesterol.

### Prolonged LDL loading of prostate cancer cells is associated with increased p38MAPK and ERK signaling

Given that prostate cancer cell growth is coupled to AR signaling, which in CRPC is linked to de novo steroidogenesis that uses cholesterol as a precursor [7], we hypothesized that LDL-inducible changes in cell proliferation of AR-positive C4-2B cells could be associated with activation of AR signaling. To test this, C4-2B cells were cultured in FCS or LPDS-containing media in the presence or absence of LDL, and then, cell lysates were analyzed for the expression of AR and PSA, as endpoint readouts of AR-regulated steroidogenesis. In lipid-lowering conditions, which is associated with reduced cellular cholesteryl ester levels (Fig. 2a) and cell proliferation (Fig. 2c and d), C4-2B expressed reduced amounts of AR and PSA proteins (Figure S4A). Upon addition of LDL, which increased cellular cholesteryl ester levels (Fig. 2a) and cell growth (Fig. 2c and d), AR and PSA protein levels remained unchanged compared to cells cultured in LPDS (Figure S4A).

As we did not observe changes in AR signaling that could explain the lipid-regulated cell proliferation patterns, we considered other growth-promoting signaling cascades that are activated by LDL, commonly after short incubation periods, including Erk1/2, p38MAPK, Akt, and G-protein pathways [31–35], which have also been implicated in prostate cancer biology [36–38]. We incubated cells with or without LDL overnight, and then used a multiplex bead-based approach to assess if prolonged cholesterol loading could alter the activity of a range of mitogenic signaling pathways. Culturing C4-2B cells in LPDS caused a reduction in PI3K/mTOR signaling and p38MAPK signaling (Figure S4B). Following prolonged LDL exposure, there was a significant increase in p38MAPK activation (Figure S4C), which aligned with increased cholesteryl ester content and cell proliferation under these conditions (Fig. 2). In addition, although only significant for MSK1, phosphorylation of the MEK1/ERK/MSK1 axis was modestly increased upon overnight LDL incubation (Figure S4B), indicating that these minor changes in activity could also contribute to enhanced proliferation.

### Inhibition of cholesteryl ester synthesis and hydrolysis blocks the ability of LDL to stimulate prostate cancer cell proliferation

**Fig. 3:**
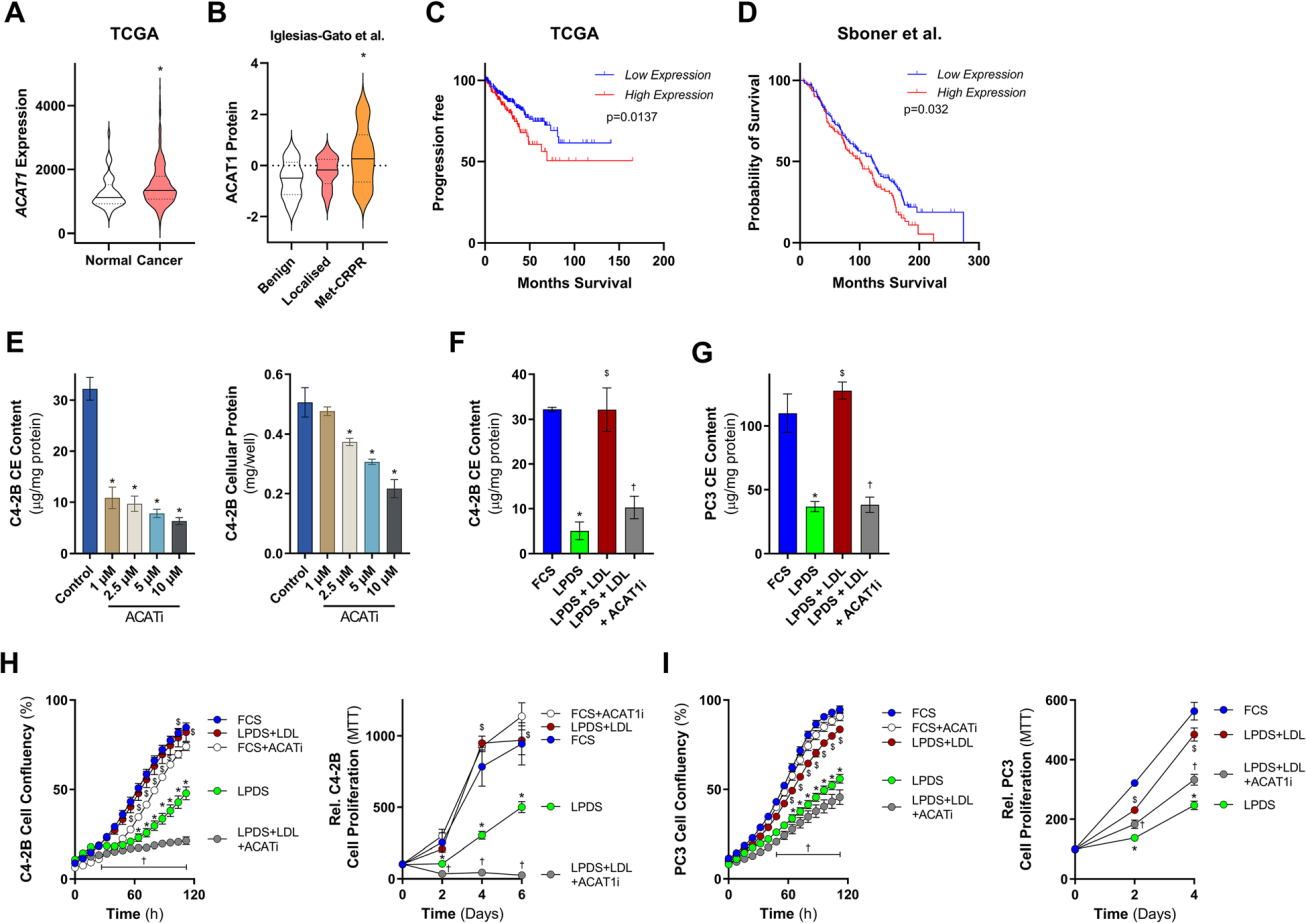
LDL supplementation rescues C4-2B and PC3 cell growth in LPDS-containing media and is blocked by inhibition of ACAT. **a** Violin plots demonstrate that *ACAT1* mRNA is overexpressed in prostate cancer tissue compared to normal in the TCGA dataset. **b** Violin plots demonstrate that ACAT1 protein level is increased in metastatic castrate-resistance prostate cancer (Met-CRPC) tissue compared to Benign prostate tissue. ACAT mRNA expression is associated with **c** shorter progression-free survival in TCGA prostate cancer dataset and **d** overall survival in Sboner et al. [57] dataset. **e** C4-2B cholesteryl ester levels and cellular protein following 24 h culturing in media supplemented with 10% FCS and increasing concentrations of the ACAT inhibitor Avasimibe. **f** C4-2B and **g** PC3 cholesteryl ester levels following 24 h culturing in media supplemented with 10% FCS, 10% LPDS, 10% LPDS plus 50 μg/ml of human LDL (LPDS+LDL), or 10% LPDS plus 50 μg/ml of human LDL and 1 μM of the ACAT inhibitor Avasimibe (LPDS+LDL+ACATi). **h** C4-2B cell proliferation cultured in media containing 10% FCS, 10% LPDS, LPDS+LDL, or LPDS+LDL+ACATi by IncuCyte and MTT. **i** PC3 cell proliferation cultured in media containing 10% FCS, 10% LPDS, LPDS+LDL, or LPDS+LDL+ACATi by IncuCyte and MTT. Data in **a** and **b** are represented as violin plots in GraphPad Prism: the horizontal line within the violin represents the median, and the dashed lines representing the quartiles. * *P* ≤ 0.05 vs. normal/benign; # *P* ≤ 0.05 vs. cancer by Mann-Whitney two-tailed *t* test (**a**) or one-way ANOVA (**b**) followed by Tukey’s multiple comparisons test. Data in **c** and **d** was analyzed using a two-sided log-rank test. Data in **e** and **i** are presented as mean ± SEM of at least three independent experiments performed in triplicate. * *P* ≤ 0.05 vs. FCS; $ *P* ≤ 0.05 vs. LPDS; † *P* ≤ 0.05 vs. LPDS+LDL by one-way ANOVA (**e**–**g**) or two-way ANOVA (**h** and **i**) followed by Tukey’s multiple comparisons test

Based on our findings that elevated cellular cholesteryl ester levels were associated with increased prostate cancer cell proliferation, we hypothesized that increased cholesteryl ester synthesis could occur in prostate cancer. This rationale is supported by the fact that *ACAT1* mRNA expression is higher in clinical prostate tumor tissue compared to normal (Fig. 3a), ACAT1 protein levels are increased in metastatic CRPC tissue compared to benign prostate tissue (Fig. 3b), and *ACAT1* mRNA expression is significantly associated with progression-free and overall survival rate (Fig. 3c and d). These observations are consistent with previous reports describing ACAT1 as a potential prognostic marker for aggressive prostate cancer [39].

To further elucidate the role of cholesteryl ester homeostasis in prostate cancer cell proliferation, we next determined whether pharmacological inhibition of ACAT1-catalyzed cholesteryl ester synthesis could block the ability of LDL to stimulate cell proliferation. Therefore, we first performed dose response experiments using the pharmacological ACAT1 inhibitor, avasimibe, and determined that 1 μM avasimibe treatment for 24 h was sufficient to reduce cholesteryl ester levels in C4-2B cells without altering cellular protein content, indicating a lack of cell toxicity under these conditions (Fig. 3e). As hypothesized, the LDL-induced increase of cholesteryl ester levels in C4-2B cells was effectively suppressed by ~70% in the presence of 1 μM avasimibe (Fig. 3f), with similar patterns observed in PC3 cells (Fig. 3g). While 1 μM avasimibe (ACAT1i) did not alter C4-2B and PC3 cell proliferation when cultured in FCS (Fig. 3h and i), ACAT1i blunted the ability of LDL to stimulate C4-2B (Fig. 3h) and PC3 (Fig. 3i) cell growth in LPDS-containing media. These observations suggest that LDL-derived cholesterol is delivered to the endoplasmic reticulum, where ACAT1-mediated cholesterol esterification occurs, followed by cholesteryl ester storage in LDs. This pool of LDL-derived cholesteryl esters in LDs are capable to support C4-2B and PC3 cell proliferation in conditions where cholesterol availability is limiting.

**Fig. 4:**
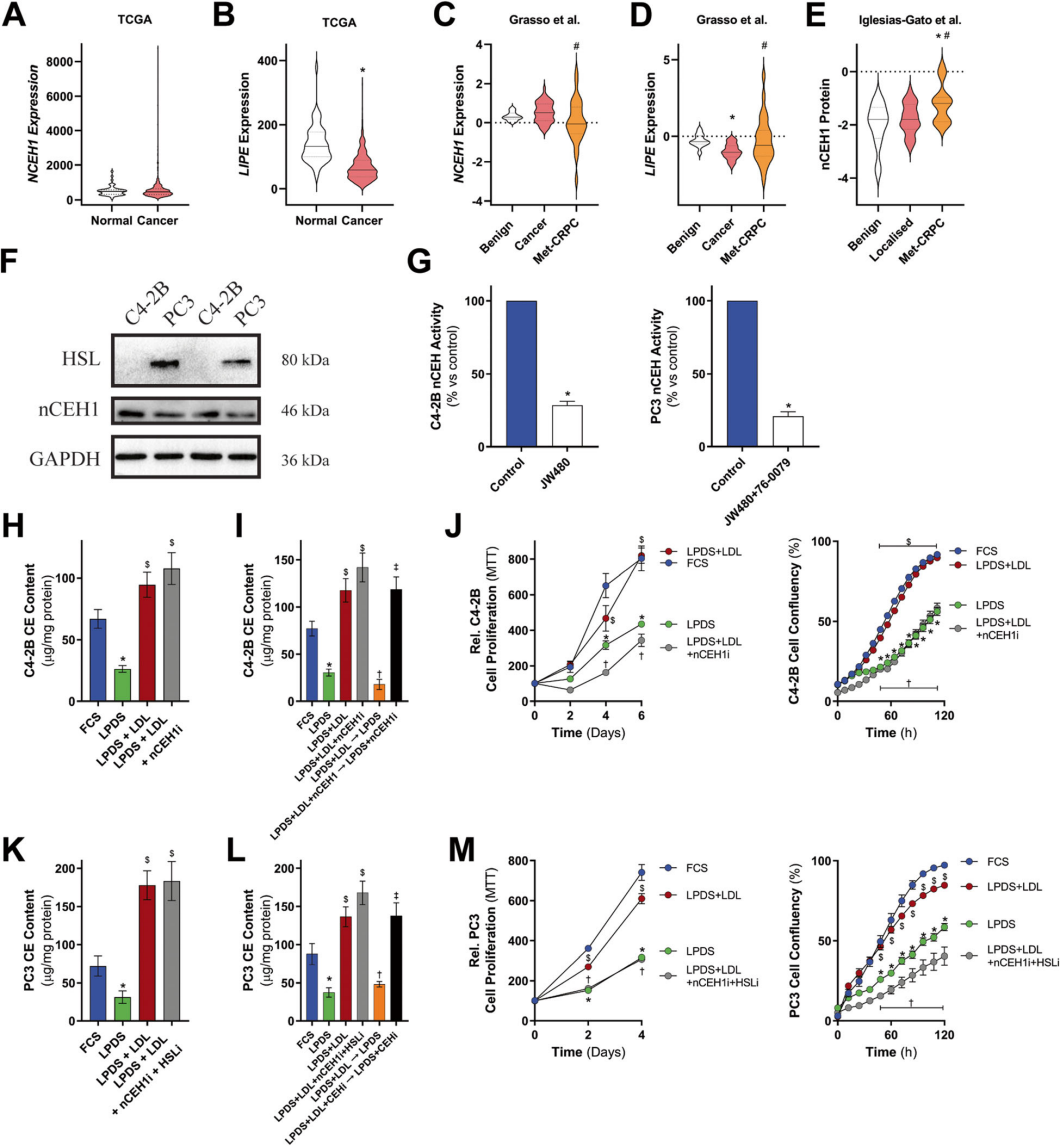
LDL supplementation rescues C4-2B and PC3 cell growth in LPDS-containing media and is blocked by inhibition of neutral cholesteryl ester hydrolysis activity. Violin plots of **a** *NCEH1* and **b** *LIPE* mRNA expression in prostate cancer tissue compared to normal in the TCGA dataset. Violin plots of **c** *NCEH1* and **d** *LIPE* mRNA benign, primary prostate cancer tissue and metastatic castrate-resistance prostate cancer (Met-CRPC) tissue. **e** Violin plots of nCEH1 protein levels in benign, primary prostate cancer tissue and metastatic castrate-resistance prostate cancer (Met-CRPC). **f** Representative immunoblots of neutral cholesteryl ester hydrolases hormone-sensitive lipase (HSL) and neutral cholesteryl ester hydrolase 1 (nCEH1) protein levels in C4-2B and PC3 cells. **g** C4-2B and PC3 neutral cholesteryl ester hydrolase (nCEH) activity of lysates from cells cultured with 1 μM of the nCEH1 inhibitor JW480 (C4-2B cells) or 1 μM JW480 and 1 μM of the HSL inhibitor 76-0079 in 10% FCS supplemented media for 2 h. **h** C4-2B cholesteryl ester levels following 24 h culturing in media supplemented with 10% FCS, 10% LPDS, 10% LPDS plus 50 μg/ml of human LDL (LPDS+LDL), or 10% LPDS plus 50 μg of human LDL and 1 μM of the nCEH1 inhibitor JW480 (LPDS+LDL+nCEH1i). **i** Pulse-Chase assessment of C4-2B cholesteryl ester turnover, where cells were either cultured for 24 h in media supplemented with 10% FCS, 10% LPDS, LPDS+LDL, or LPDS+LDL+nCEH1i, then cells cultured in LPDS+LDL or LPDS+LDL+nCEH1i were then cultured in either LPDS or LPDS+nCEH1i. **j** C4-2B cell proliferation cultured in media containing 10% FCS, 10% LPDS, LPDS+LDL, or LPDS+LDL+nCEH1i by IncuCyte and MTT. **k** PC3 cholesteryl ester levels following 24 h culturing in media supplemented with 10% FCS, 10% LPDS, 10% LPDS plus 50 μg/ml of human LDL (LPDS+LDL), or 10% LPDS plus 50 μg of human LDL and 1 μM of the nCEH1 inhibitor JW480 and 1 μM of the HSL inhibitor 76-0079 (LPDS+LDL+nCEH1i+HSLi). **l** Pulse-Chase assessment of PC3 cholesteryl ester turnover, where cells were either cultured for 24 h in media supplemented with 10% FCS, 10% LPDS, LPDS+LDL, or LPDS+LDL+nCEH1i, then cells cultured in LPDS+LDL or LPDS+LDL+nCEH1i+HSLi were then cultured in either LPDS or LPDS+nCEH1i+HSLi. **m** PC3 cell proliferation cultured in media containing 10% FCS, 10% LPDS, LPDS+LDL, or LPDS+nCEH1i+HSLi by IncuCyte and MTT. Data in **a** and **b** are represented as violin plots in GraphPad Prism: the horizontal line within the violin represents the median, and the dashed lines representing the quartiles. * *P* ≤ 0.05 vs. normal/benign; # *P* ≤ 0.05 vs. cancer by Mann-Whitney two-tailed *t* test (**a** and **b**) or one-way ANOVA (**c**e) followed by Tukey’s multiple comparisons test. Data in **g**–**m** are presented as mean ± SEM of at least three independent experiments performed in triplicate. * *P* ≤ 0.05 vs. FCS; $ *P* ≤ 0.05 vs. LPDS; † *P* ≤ 0.05 vs. LPDS+LDL; ‡ *P* ≤ 0.05 vs. LPDS+LDL+nCEH1i(+HSLi) by one-way ANOVA (**g**–**i**, **k** and **l**) or two-way ANOVA (**j** and **m**) followed by Tukey’s multiple comparisons test

The requirement for cholesterol esterification to support prostate cancer cell proliferation implicates an important role for LD-contained cholesteryl esters. As such, we hypothesized that the release of cholesterol from LDs would be required to promote prostate cancer cell proliferation. We therefore first addressed the potential clinical relevance of this hypothesis. While the precise coordination of cholesteryl ester breakdown remains to be clarified, nCEH1 (also known as AADACL1 and KIAA1363) and HSL have been reported to exhibit neutral cholesterol ester hydrolase activity [40, 41]. *NCEH1* mRNA expression was not different between normal and cancer tissue (Fig. 4a), whereas *LIPE* mRNA (which encodes HSL) expression was reduced in cancer tissue (Fig. 4b). *NCEH1* mRNA expression was lower whereas *LIPE* mRNA expression was higher in metastatic prostate cancer tissue compared to primary tissue (Fig. 4c and d); however, the protein levels of nCEH1 were increased in metastatic CRPC tissue compared to benign and primary cancer tissue, while HSL protein was not reported/detected (Fig. 4e). We were unable to determine a consistent association between *NCEH1* or *LIPE* expression and progression-free or overall patient survival (Figure S5).

We next examined the consequences of blocking cholesterol release from cholesteryl ester-containing LDs, by targeting cholesteryl ester hydrolase activity. Western blot analysis revealed that C4-2B cells expressed nCEH1 but not HSL, whereas PC3 cells expressed both nCEH1 and HSL (Fig. 4f). Using an in vitro assay of neutral cholesterol ester hydrolase activity (Figure S6) and supporting the expression patterns observed in these two cell lines, C4-2B neutral cholesterol ester hydrolase activity was reduced in the presence of 1 μM nCEH1 inhibitor, JW480, and the combination with 1 μM HSL inhibitor, 76-0079, reduced PC3 neutral cholesterol ester hydrolase activity (Fig. 4g). Cholesteryl ester levels were lower in C4-2B and PC3 cells cultured in LPDS-containing media compared to cells grown in FCS. Addition of LDL restored cholesteryl ester levels in LPDS-containing media, but there was no further increase with nCEH1 inhibition (Fig. 4h).

These findings indicated a limited ability of the neutral cholesterol ester hydrolase inhibitor to further increase cholesteryl ester levels at steady state conditions, in the presence of incoming LDL-cholesterol. To measure neutral cholesterol ester hydrolase activity more directly, we therefore assessed cholesteryl ester turnover in LDL-loaded cells in the presence of nCEH1 inhibitor using a pulse-chase approach. C4-2B cells were cultured in LPDS+LDL for 24 h, then chased with LPDS-containing media with or without nCEH1 inhibitor. We observed that LPDS+LDL for 24 h increased cholesterol ester levels, which were then reduced in cells cultured in LPDS media, whereas nCEH1 inhibition maintained high levels of cholesteryl ester (Fig. 4i). Strikingly, the change in cholesteryl ester turnover correlated with altered cell growth. Specifically, the ability of LDL to promote C4-2B cell growth was blocked by inhibition of nCEH1 (Fig. 4j). Similarly, inhibiting both nCEH1 and HSL in PC3 cells did not further increase cholesteryl ester levels compared to LDL supplementation (Fig. 4k) but did blunt cholesteryl ester turnover (Fig. 4l). Moreover, inhibition of neutral cholesteryl ester hydrolysis in PC3 cells blocked the ability of LDL to promote cell growth (Fig. 4m). Further, similar patterns of neutral cholesteryl ester hydrolysis-sensitive cholesteryl ester content and cell growth were observed when performed in CS-LPDS (Figure S7). As such, the ability of LDL to stimulate cell growth requires delivery of LDL-derived cholesterol to LDs and subsequent mobilization of stored cholesterol through cholesteryl ester hydrolase activity.

### Inhibition of cholesteryl ester hydrolysis blunts C4-2B and PC3 cell proliferation in charcoal-stripped media

**Fig. 5:**
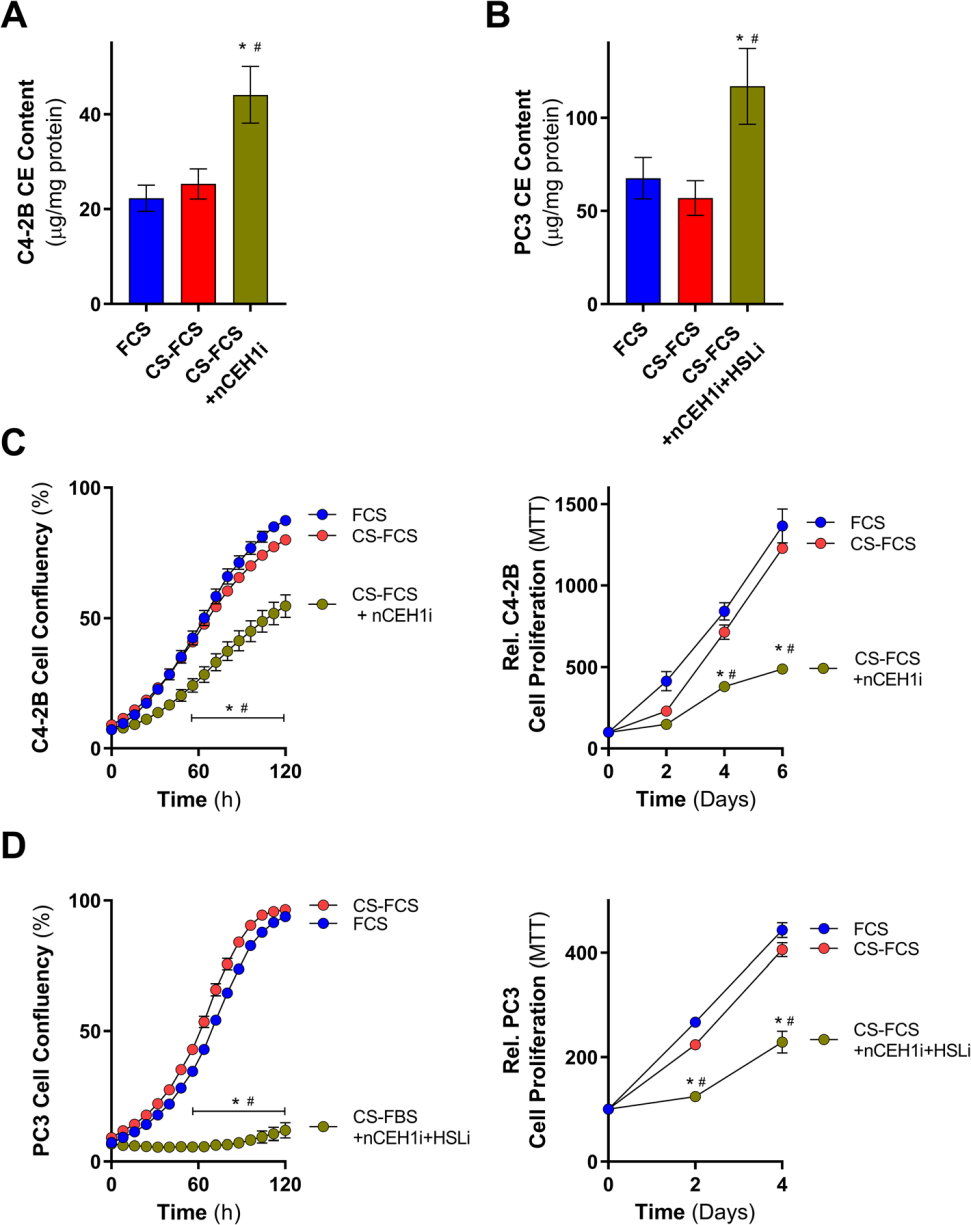
Inhibition of neutral cholesteryl ester hydrolysis activity slows C4-2B and PC3 cell growth in CS-FCS-containing media. **a** C4-2B cholesteryl ester levels following 24 h culturing in media supplemented with 10% FCS, 10% CS-FCS, and 10% CS-FCS plus 1 μM of the nCEH1 inhibitor JW480 (CS-FCS+nCEH1i). **b** PC3 cholesteryl ester levels following 24 h culturing in media supplemented with 10% FCS, 10% CS-FCS, 10% CS-FCS plus 1 μM of the nCEH1 inhibitor JW480, and 1 μM of the HSL inhibitor 76-0079 (CS-FCS+nCEH1i+HSLi). **c** C4-2B cell proliferation cultured in media containing 10% FCS, 10% CS-FCS, or CS-FCS+nCEH1i+HSLi by IncuCyte and MTT. **d** PC3 cell proliferation cultured in media containing 10% FCS, 10% CS-FCS, or CS-FCS+nCEH1i+HSLi by IncuCyte and MTT. Data are presented as mean ± SEM of at least three independent experiments performed in triplicate. * *P* ≤ 0.05 vs. FCS; # *P* ≤ 0.05 vs. CS-FCS by one-way ANOVA (**a** and **b**) or two-way ANOVA (**c** and **d**) followed by Tukey’s multiple comparisons test

We next examined if the sustained prostate cancer cell growth in a low androgen environment requires neutral cholesterol ester hydrolase activity and the hydrolysis of cholesteryl esters. As such, we tested whether inhibition of cholesteryl ester hydrolysis influenced the growth of androgen-independent C4-2B and PC3 cells in low androgen growth condition, using charcoal-stripped FCS. Inhibition of nCEH1 increased cholesteryl ester levels in C4-2B cells cultured in CS-FCS compared to cells cultured in FCS and CS-FCS (Fig. 5a). Likewise, inhibition of both nCEH1 and HSL increased cholesteryl ester levels in PC3 cells cultured in CS-FCS compared to cells cultured in media containing FCS and CS-FCS (Fig. 5b). Importantly, pharmacological inhibition of cholesteryl ester hydrolysis was associated with strongly reduced C4-2B (Fig. 5c) and PC3 (Fig. 5d) cell growth in CS-FCS. The turnover of cholesteryl esters therefore appears critical for prostate cancer cell growth in a low androgen environment, even when the supply with extracellular cholesterol is not limiting.

## Discussion

**Fig. 6:**
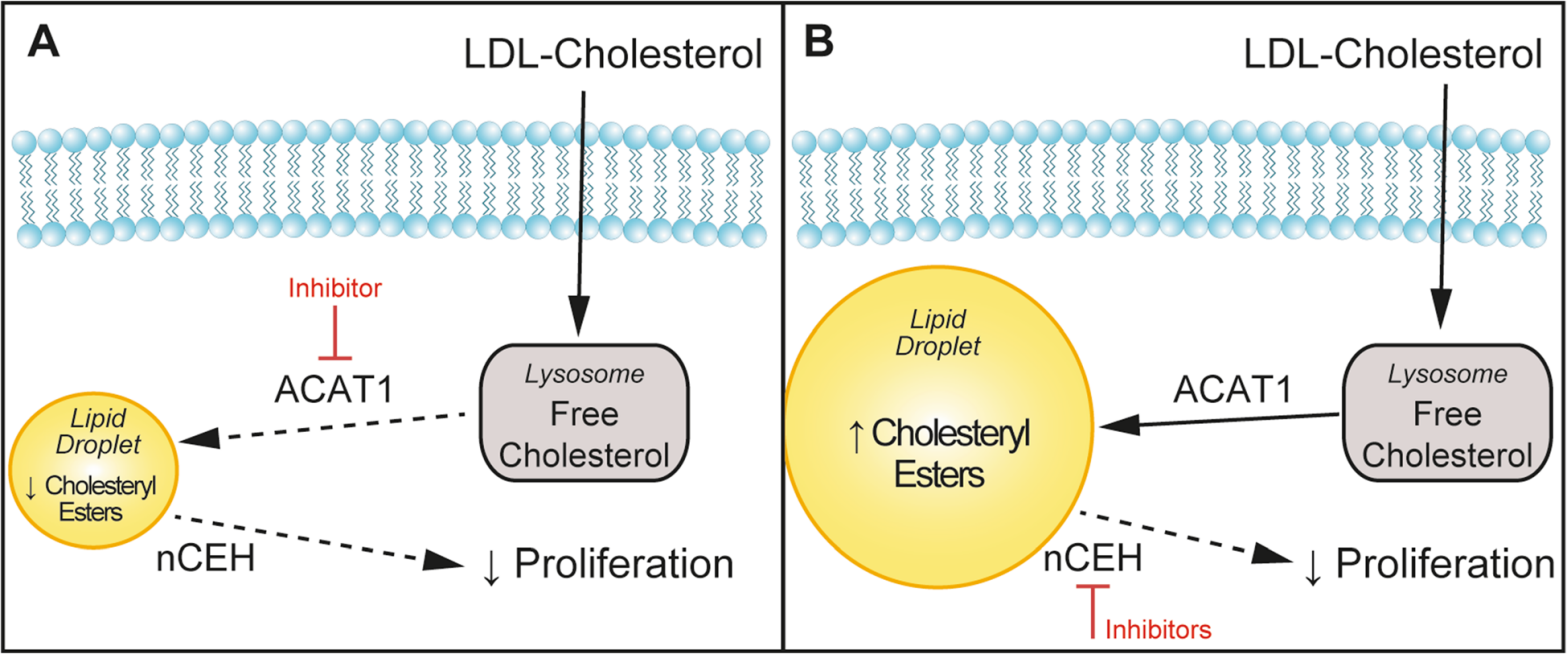
The relationship between LDL-cholesterol metabolism, cholesteryl ester turnover, and proliferation of prostate cancer cell proliferation. The schematic shows that prostate cancer cell proliferation is reduced by pharmacological inhibition of **a** ACAT1 to block cholesterol esterification and reduced cholesteryl ester levels, or **b** neutral cholesteryl ester hydrolase (nCEH) activity which increased cholesteryl ester levels

Cholesterol is an essential constituent of cellular membranes and is therefore a requisite for cancer cell growth [42]. An emerging feature of cancer cell progression is the role that extracellular nutrient availability plays and the evidence of metabolic adaptability, which consists of flexibility in substrate utilization as well as metabolizing substrates in different ways [43]. This is exemplified by clinical observations linking hypercholesterolemia and prostate cancer progression [6, 8–14]; however, the importance of extracellular LDL-cholesterol and cholesteryl ester homeostasis in prostate cancer progression has not been fully elucidated. Using cell culture and a range of selectively modified sera, we demonstrate that reducing lipoprotein availability in the media reduced C4-2B (AR-positive, androgen-independent) and PC3 (AR-negative, androgen-independent) cell cholesteryl ester levels and cell growth. This reduced cell growth of C4-2B and PC3 was recovered by supplementation of exogenous LDL and required cholesterol esterification as well as cholesteryl ester hydrolysis activity (Fig. 6). This suggests that uptake of extracellular cholesterol, through endocytosis of LDL-derived cholesterol and subsequent delivery and storage in the LD-contained cholesteryl ester pool, is required to support prostate cancer growth. Interestingly, the prolonged exposure of prostate cancer cells with LDL and the continuous accumulation of cholesteryl esters are associated with several changes in cellular signaling events, as assessed by the multiplex assessment of 20 signaling intermediates. These findings indicate that LDL- or cholesteryl ester-induced mechanism(s) linking cholesterol metabolism and proliferation may occur at early incubation times, followed by commonly observed signal downregulation for several signaling cascades. Yet, other signaling modules are elevated even after prolonged LDL-loading periods and possibly contribute to triggering continuous upregulation of biochemical pathways involved in cholesterol homeostasis that are required to support cell growth. These observations highlight a central role for cholesterol esterification and cholesteryl ester hydrolysis in prostate cancer cell biology that warrants further mechanistic investigation in suitable in vivo models to design strategies for targeting these aspects of cholesterol metabolism in androgen-independent prostate cancer.

The cellular free cholesterol pool has several inputs, including de novo synthesis, hydrolysis of cholesteryl esters, and uptake of extracellular sources such as LDL. In prostate cancer, the feedback loops between these pathways is disrupted, leading to increased amounts of LDLR at the cell surface and thereby elevating uptake of LDL-derived cholesterol, altogether raising cellular cholesterol levels [16]. Several studies have demonstrated clear links between circulating cholesterol levels and prostate cancer biology, including hypercholesterolemia and more aggressive disease [9–12], and conversely lowering cholesterol by statin usage reduces prostate cancer-specific mortality [13, 14]. Similar observations have been made in other cancers, including breast cancer [44–46]. Additionally, hypercholesterolemic diet feeding promotes tumor growth in the in vivo LNCaP xenograft model [47]. Here, we showed an intimate dependency between extracellular availability of lipids, especially LDL-cholesterol, and androgen-independent prostate cancer cell growth. Importantly, this was observed in normal and reduced testosterone levels and in AR-positive and AR-negative cells. Similar studies have shown that LDL supplementation can increase PC3 and LNCaP prostate cancer cell line proliferation [17, 48]. While these cell culture studies were performed in FCS-containing media, which contains cholesterol-rich lipoproteins, we now show for the first time that LDL supplementation of LPDS-containing media restores cell growth. LDLR levels have been reported to be elevated in prostate cancer, especially metastatic disease [49] but a recent study reported that the scavenger receptor B1 (SR-B1, encoded by *SCARB1*), which rather acts as a receptor for high-density lipoproteins (HDL), and not LDLR is upregulated [50]. Further, the same study also showed that loss of function or pharmacological antagonism of SR-B1 reduced HDL uptake and slowed growth of a range of prostate cancer cells [50]. Collectively, these observations demonstrate that extracellular cholesterol levels influence prostate cancer cell growth in a range of settings.

Prostate cancer cell growth is coupled to AR signaling [51]. One adaptive mechanism that overcomes reduced systemic availability of androgen, leading to the development of CRPC, is the intracellular activation of de novo steroidogenesis that ultimately (re-)activates AR signaling [7]. Steroidogenesis requires cholesterol as its starting substrate, which in steroidogenic tissues is sourced from cholesteryl ester-rich LDs that accumulate in these tissues [18]. Prostate cancer tissues also accumulate LDs that are cholesteryl ester-rich, and there is further increased LD accumulation in high-grade localized prostate cancer and metastatic cancer compared to low-grade localized cancer [17]. These combined observations suggested that the effect of cholesterol availability on prostate cancer cell proliferation could be linked to AR signaling via de novo steroidogenesis. In our study, we observed that cholesteryl ester levels were indeed sensitive to extracellular cholesterol availability but, unexpectedly, LDL supplementation of LPDS-containing media, which restored cell growth to rates similar to cells cultured in FCS, was not accompanied by activation of AR signaling. Therefore, we measured other known growth-promoting signaling cascades, including Erk1/2, p38MAPK, Akt, and G-protein pathways [31–35]. In this analysis, we observed that PI3K/mTOR signaling and p38MAPK signaling was reduced in C4-2B cells cultured in LPDS-containing media and that p38MAPK signaling and MEK1/ERK/MSK1 axis was activated in cells cultured in LDL-supplemented LPDS media. A number of studies have shown that several protein kinases known to stimulate cell proliferation, including Erk1/2, p38MAPK, Akt, mTORC1, and heterotrimeric G-proteins, are activated in response to LDL exposure [31–35, 52]; however, these studies used much shorter exposure times (5 min–6 h) than we used [24 h]. Since we observed changes in cholesteryl ester levels after 24 h and cell proliferation after 48h of culturing in modified media, we hypothesized that meaningful changes in AR signaling as a consequence of the intracellular production of androgens from LDL-cholesterol and/or LD-cholesterol would be evident at 24 h. Ultimately, our data indicate that LDL-stimulated cell proliferation was associated with sustained activation (i.e., phosphorylation) of p38MAPK and MSK1. It is possible that moderate changes in the phosphorylation status of other protein kinases observed after prolonged LDL incubation periods that formed the basis of cholesteryl ester measurements and proliferation kinetics do not fully reflect the signaling events that occur at earlier time points possibly leading to molecular signals that further links extracellular lipid levels to cell proliferation rates.

The esterification of free cholesterol at the endoplasmic reticulum by ACAT1 and storage in LDs protects against the unnecessary built up of free cholesterol within cell membranes [53]. Increased abundance of cholesteryl ester-rich LDs associates with increased prostate cancer aggressiveness [17]. The outcomes of our experiments demonstrate that intracellular cholesteryl ester levels are influenced by extracellular lipid levels and associates with prostate cancer cell proliferation. Consistent with Yue and colleagues [17], pharmacological inhibition of ACAT1 reduced both cholesteryl ester levels and cell proliferation, which further adds to the growing body of literature promoting the potential for ACAT1 as an anti-cancer therapeutic target (see reviews [54, 55]). Further, elevated ACAT1 expression is associated with reduced time to biochemical recurrence of prostate cancer [56] as well as progression-free and overall survival. In our experiment, ACAT1 inhibition blocked the ability of LDL supplementation in LPDS media to rescue cell proliferation, whereas Yue et al. performed experiments in cholesterol-rich FCS [17]. In addition, while sub-IC_50_ concentrations inhibited ACAT1 (1 μM avasimibe)-dependent cholesterol esterification and LDL-cholesterol mediated rescue of cell growth in LPDS-containing media in our studies, Yue and coworkers examined PC3 cells in the presence of IC_50_ concentrations of avasimibe (7.5 μM). Strikingly, our results also demonstrate that inhibition of cholesteryl ester hydrolysis (i.e., breakdown) to mobilize stored cholesterol and fatty acids blocked the ability of LDL-cholesterol supplementation to restore growth of cells cultured in LPDS media. Additionally, cholesteryl ester hydrolysis supports prostate cancer cell growth in low androgen environments, which is CS-FCS. These results somewhat challenge the notion that the growth-inhibitory effects of blocking cholesteryl ester homeostasis are due to excess free extracellular cholesterol availability and therefore efforts to downregulate LDLR and essential fatty acid uptake [17]. The fact that inhibiting cholesteryl ester hydrolysis reduced the proliferation of cells cultured in LDL-supplemented LPDS implicates critical growth promoting additional mechanisms downstream of LDL endocytosis and cholesterol esterification. As such, cellular cholesteryl ester homeostasis influences prostate cancer cell growth and inhibition of cholesteryl ester hydrolysis may provide novel opportunities to suppress prostate cancer progression.

## Conclusion

This study has identified extracellular lipid levels and LDL-cholesterol availability and cholesteryl ester metabolism at the LD, especially neutral cholesteryl ester hydrolysis, as important processes supporting androgen-independent prostate cancer cell growth. These novel insights advance our understanding of the mechanisms that link hypercholesterolemia with more aggressive prostate cancer. Given that our findings are based on cell culture models, additional preclinical evidence demonstrating these mechanisms in androgen deprivation and hypercholesterolemic conditions that characterize late-stage disease is warranted to consider targeting LD-associated cholesterol metabolism in androgen-independent prostate cancer.

## Data Availability

All data generated or analyzed during this study are included in this published article and its supplementary information files.

## Abbreviations

ACAT1: Acyl-Coenzyme A: cholesterol acyltransferase 1
AR: Androgen receptor
CE: Cholesteryl ester
CRPC: Castrate-resistant prostate cancer
CS-FCS: Charcoal-stripped fetal calf serum
CS-LPDS: Charcoal-stripped lipoprotein-deficient fetal calf serum
ERK: Extracellular signal-regulated kinase
FCS: Fetal calf serum
GAPDH: Glyceraldehyde 3-phosphate dehydrogenase
GEO: Gene Expression Omnibus
HSL: Hormone-sensitive lipase
LD: Lipid droplet
LDL: Low-density lipoprotein
LDLR: Low-density lipoprotein receptor
LPDS: Lipoprotein-deficient fetal calf serum
MAPK: Mitogen-activated protein kinase
nCEH1: Neutral cholesteryl ester hydrolase 1
PSA: Prostate-specific antigen
TCGA: The Cancer Genome Atlas
